# Type 2 Diabetes—Effect of Compensatory Oversecretion as a Reason for β-Cell Collapse

**DOI:** 10.1080/15604280214276

**Published:** 2002

**Authors:** Valdemar Grill, Anneli Björklund

**Affiliations:** 1 Department of Internal Medicine Medical Faculty Norwegian University of Science and Technology Trondheim Norway; 2 Department of Molecular Medicine Karolinska Institute Stockholm Sweden; 3 Department of Medicine University Hospital of Trondheim Trondheim 7006 Norway

## Abstract

Insulin secretion declines progressively before and during the course of type 2 diabetes. Evidence indicates that this process is, in part, secondary to increased requirement for insulin secretion that is brought about by insulin resistance and by hyperglycemia. The effects of over-secretion extend far beyond a mere reduction of available insulin stores and may cause not only functional but also structural damage. The time is ripe for clinical studies, which explore the therapeutic potential of reducing over-secretion.


*Int. Jnl. Experimental Diab. Res.*, 3:153–158, 2002
Copyright © 2002 Taylor & Francis
1560-4284/02 $12.00 + .00
DOI: 10.1080/15604280290013919

Taylor & Francis healthsciences

**Review**

### Type 2 Diabetes—Effect of Compensatory Oversecretion as a Reason for β-Cell Collapse

**Valdemar Grill[1,2] and Anneli Björklund[2]**

[1]*Department of Internal Medicine, Medical Faculty, Norwegian University of Science and Technology, Trondheim Norway*
[2]*Department of Molecular Medicine, Karolinska Institute, Stockholm, Sweden*

**Insulin secretion declines progressively before and during the course of type 2 diabetes. Evidence indicates that this process is, in part, secondary to increased requirement for insulin secretion that is brought about by insulin resistance and by hyperglycemia. The effects of over-secretion extend far beyond a mere reduction of available insulin stores and may cause not only functional but also structural damage. The time is ripe for clinical studies, which explore the therapeutic potential of reducing over-secretion.**

**Keywords** Beta cell; Diazoxide; Insulin Secretion; Type 2 Diabetes

Insulin release is deficient in Type 2 diabetes. Genetic causes of deficient insulin secretion in Type 2 diabetes are well recognized [1, 2]. However, accumulating evidence indicates that non-genetic factors are also at play, some of which are linked to the metabolic abnormalities evolving with, or preceding diabetes. That such factors are important is suggested by the fact that insulin deficiency worsens with the duration of diabetes [3, 4], such worsening being the major reason for deterioration of metabolic control and the need for insulin treatment that evolves in the course of the disease. Furthermore, a number of studies have shown that adequate treatment of diabetes has beneficial effects on insulin secretion [5–9].

Chronic hyperglycemia and dyslipidemia are two metabolic abnormalities that are proposed to negatively affect pancreatic beta cells [10]. The negative effects are often referred to respectively as "glucotoxicity" and "lipotoxicicty". The importance of "glucotoxicity" is by now well established whereas that of "lipotoxicity" is still under debate.

The mechanisms whereby chronic hyperglycemia affects beta cell functions have not been fully elucidated. By analogy with the role of glucose in diabetic complications, the glucose molecule could be thought to interact directly with proteins or other molecules producing glycation products [11] and could be metabolized to products which cause oxidative stress [12]. Indeed, evidence has been forthcoming to that extent [12–14]. However, since the beta-cells are primarily engineered to respond to even small elevations of glucose above fasting levels, the possibility exists that chronic hyperglycemia exerts negative effects by enforcing a state of oversecretion in beta cells. Evidence, to be reviewed here, indicates that such "beta cell exhaustion" is harmful to beta cell function and perhaps survival. It follows that other conditions which separately or in concert with hyperglycemia cause oversecretion could also be harmful. Such conditions include insulin resistance and pharmacological treatment with sulphonylurea compounds.

In the following, we will review some of the evidence for an important role of oversecretion behind deficient and dysregulated insulin release in diabetes. Most, but not all, of this evidence comes from animal studies. Possible mechanisms behind oversecretion will be discussed and possible strategies to avert oversecretion will be presented.

### ANIMAL STUDIES: OVERSECRETION BY HYPERGLYCEMIA RAPIDLY DESENSITIZES BETA CELLS TO GLUCOSE

In 1986, Leahy, et al. reported that 48 h of marked hyperglycemia in normal rats, achieved by massive glucose infusions,

The authors' studies have been supported by the Norwegian and Swedish Research Councils, and by the Swedish Diabetes Association.
Address correspondence to Valdemar Grill, Department of Medicine, University Hospital of Trondheim, 7006 Trondheim, Norway. E-mail: valdemar.grill@medisin.ntnu.no produced almost total insensitivity to glucose when insulin release was subsequently measured from perfused pancreas [15]. The desensitizing effect was specific for glucose, other secretagogues, such as arginine exerting normal or even exaggerated responses. One of us (V.G.) later showed that if glucose-induced insulin secretion during the glucose infusion was blocked by the simultaneous infusion of diazoxide, then no desensitization occurred; if anything, insulin responses to glucose were enhanced [16]. Since the levels of hyperglycemia were kept the same in experiments with or without diazoxide, it was concluded that the desensitizing effect was not due to any effects of hyperglycemia per se. We considered the possibility that the decrease in hyperinsulinemia brought about by diazoxide could be important. Indeed, the addition of a continuous insulin infusion to the protocol with diazoxide diminished glucose-induced insulin secretion [16]. However, in vitro studies with pancreatic islets cultured overnight with diazoxide at low and high glucose reproduced the beneficial effects of diazoxide but did not reveal any effect of exogenously added insulin during culture [17]. Therefore we concluded that beta cell over-secretion was the cause of desensitization to glucose.

Studies in another animal model, the 90% pancreatectomized rat have also used diazoxide as a probe for assessing oversecretion [18]. Also in this animal model did the results indicate a profound influence of over-secretion on beta cell function.

### ANIMAL STUDIES: OVERSECRETION BY HYPERGLYCEMIA PRODUCES LASTING EFFECTS ON BETA CELL FUNCTION

The desensitizing effects of up to 48 h of hyperglycemia were shown to be reversible within 24-h [15]. The question arises whether chronic over-stimulation over months and years would produce more lasting effects. In support of this notion, we obtained evidence for a lasting effect of diazoxide treatment on B-cell function in a rat transplantation model [19]. Islets from normal rats were transplanted under the kidney capsule to syngeneic recipients previously made diabetic by streptozotocin. Diazoxide treatment of these rats for 8 weeks improved transplant function in terms of arginine-induced insulin secretion not only during, but also when tested one week after treatment, implying that over-stimulation had permanently damaged the transplanted B-cells.

### THE GK RAT

The GK rat is a non-obese model of type 2 diabetes in which deficient insulin secretion is at least the major cause of diabetes [20]. Culture of pancreatic islets from these animals with diazoxide failed to restore a normal insulin secretion, despite a resulting increase in intra-islet insulin contents [21]. Also insulin treatment at early age did not improve insulin secretion at later age [22]. The most likely explanation for these findings is that the genetic determinants of deficient insulin secretion are strong enough to override any effect of modulating the demands for insulin secretion.

### HUMAN STUDIES IN VITRO

In human pancreatic islets we found that 48-h of tissue culture at a high glucose concentration completely desensitized glucose-induced insulin secretion [23] and that this desensitization could be corrected by co-culture with diazoxide. We also demonstrated that over-secretion is coupled to a preferential increase of proinsulin release and presence in the islets. Culture of human pancreatic islets for 48 h at 27 mM glucose thus markedly increased the ratio of immunoreactive proinsulin to insulin in islets as well as in secreted products both during and after culture [23]. Furthermore, the increased ratio both of stored and secreted products was completely normalized by blocking insulin secretion with diazoxide. These results are analogous to those in rat pancreas of 90% pancreatectomized rats, receiving or not receiving diazoxide [24].

### HUMAN STUDIES IN VIVO

As previously mentioned, there are many studies in type 2 diabetic patients which demonstrate that correction or at least amelioration of hyperglycemia can improve insulin secretion [5–9]. None of these studies however were designed to separately evaluate oversecretion vs. other effects. Therefore, one cannot a priori deduce the participation of oversecretion or relief thereof. Only one published study using glucose infusions and somatostatin to suppress endogenous insulin secretion attests to a beneficial effect of avoiding oversecretion [25]. However, our observations in human islets that oversecretion in vitro is associated with preferential release and storage of proinsulin could render proinsulin or ratios of proinsulin to insulin in plasma a valid marker of oversecretion. It is well known that a preferential increase of proinsulin in plasma is a hallmark of type 2 diabetes and that these parameters vary with the degree of metabolic control [26] and strain on the beta cell [27]. The validity of proinsulin being a marker for oversecretion is strengthened by a large population-based epidemiological study [28] in which over 3000 subjects were screened for glucose tolerance insulin secretion, insulin resistance and proinsulin. The results of the study negated any role of genetics behind levels of proinsulin. Furthermore, the study showed a tight correspondence between the degree of insulin resistance and proinsulin levels.

Diazoxide has been tested in an open short term study in type 2 diabetes with promising effects on beta cell function [29]; however, a follow-up study reported that the drug caused insulin resistance [30]. Such resistance was not encountered in a study in newly diagnosed subjects with type 1 diabetes [31]. Subjects were treated for 3 months with placebo or with diazoxide 4–6 mg/kg body weight in divided doses. Endogenous insulin secretion, as measured by C-peptide glucagon tests was better preserved in diazoxide-treated than in placebo-treated subjects three times up to one year after the end of diazoxide treatment. Thus, the treatment effect was considerable. However, in the previous studies side effects of diazoxide (edema, hair growth, hypotension, and nausea) have been considerable. These side effects are not serious and are reversible. However, they are disturbing enough to impede further long-term studies.

### OVERSECRETION AND INSULIN RESISTANCE

Insulin resistance increases demands for insulin secretion, adding to the demands of hyperglycemia. The question arises whether insulin resistance per (or in the presence of only minimal hyperglycemia) could have harmful effects linked to beta cell oversecretion. Certainly, longitudinal data in type 2 diabetes, for instance in Pima Indians [32] are compatible with such a notion, insulin resistance preceding at least a major deficiency in insulin secretion. However, again it is not possible from such studies to deduce any specific influence of oversecretion vis-à-vis other influences. Also compatible with harmful effects of oversecretion are epidemiological data in Pima Indians showing that the duration of obesity (a marker of insulin resistance) is a risk factor for diabetes and low insulin secretion [33]; such was also the case in a Swedish study [34].

### OVERSECRETION AND DRUGS

If over-secretion is bad for the beta cell, then the use of sulphonylurea in treatment of type 2 diabetes patients may be questioned. Indeed, in vitro evidence from beta cells demonstrate negative effects of prolonged exposure to SU [35]. However, putative negative effects of SU during long term treatment on insulin secretion have so far not been specifically investigated. in man. To evaluate any such effect would require that one takes into consideration any change in metabolic control which could per se alter insulin secretion.

In order to specifically examine long term effects of SU on insulin secretion in man we randomized newly diagnosed type 2 diabetic patients to either glibenclamide or insulin treatment [36]. Insulin release as measured from C-peptide glucagon tests was significantly better in the insulin-treated group after one year of the study but not significantly so after 2 years of the study. Later follow-ups will determine whether the two treatments differ in the long run in terms of preservation of beta cell function.

### MECHANISMS BEHIND EFFECTS OF OVERSECRETION

In previous studies one usually finds a reduction of insulin contents in islets or perfused pancreas, which is concomitant with desensitization. Therefore, it has been proposed that insulin depletion fully explains the decrease in glucose-induced insulin secretion [37], in which case the term “desensitization” may not be appropriate for the phenomenon. However, with regard to studies with diazoxide, several observations indicate that the protective effects of the drug on desensitization are only partly explained by preservation of insulin contents. In this context, cooling experiments in rat islets are instructive. Cooling below 30 degrees Celsius inhibits exocytosis of insulin but only marginally decreases glucose-induced $Ca^{2+}$ inflow during glucose stimulation [38]. We found that cooling during glucose stimulation, while blocking insulin secretion and upholding islet insulin contents, only partially protected against desensitization [17]. Only after exclusion of $Ca^{2+}$ in the incubation media did cooling completely protect against desensitization. These results support the notion that persistent inflow of $Ca^{2+}$—and/or cellular events following that inflow but distinct from exocytosis—is negative for B cell functions and participates in the desensitization due to over-stimulation.

Further support for effects not linked to insulin depletion comes from recent experiments in a diabetic transplantation model. The model is similar to the one described above, except that the graft-bearing recipients were moderately rather than severely diabetic. Treatment with diazoxide for 8 weeks was followed by one week of no treatment. Grafts from rats previously receiving diazoxide were then significantly more responsive both to glucose and to nonnutrient secretagogues than grafts from placebo-treated rats, whereas insulin contents were comparable (unpublished observations). In this context it is worthy of note that insulin secretion in type 2 diabetes is reduced far out of proportion to any reduction in insulin content or beta cell mass [39].

Deposition of amyloid in pancreatic islets could be pathogenetically important in type 2 diabetes. It is well known that amyloid accumulates in type 2 diabetic subjects [40]. It probably interferes with normal beta cell function. Animal studies indicate that an influence of hyperglycemia on amyloid deposition is indirect. For example, animals with glukokinase mutations develop diabetes because of inability of elevated glucose to activate the enzyme, which in turn blocks the glucose signal for insulin secretion. These animals in whom hyperglycemia is caused by under-secretion from a normal number of beta cells seem protected from the formation of amyloid [41].

### RELEVANCE OF DIAZOXIDE AS A PROBE FOR TESTING OVERSECRETION

As evident from the fore going, much although not all, evidence for the importance of over-secretion comes from studies using diazoxide. The question arises whether drug effects that are not related to protection from oversecretion could also come into play. The inhibitory effect of diazoxide on insulin secretion stems from the activation of the drug of ATP-dependent potassium channels in the cell membrane of beta cells [42]. These effects are the opposite of those of glucose and thereby counteract the stimulating effects of glucose on insulin secretion. Diazoxide was chosen as a probe for oversecretion, because a) its inhibitory effects on insulin secretion are rapidly reversible, b) the drug has been used in clinical medicine without serious toxicity, c) the inhibitory effects during the presence of diazoxide are more pronounced than the effects of other inhibitors, such as somatostatin and adrenaline. In our hands we did not observe any beneficial effects of diazoxide on beta cell function during non-stimulatory conditions, i.e. after the presence of the drug during low-glucose conditions. Nevertheless, some effects of the drug per se cannot be wholly excluded. In this context it should be mentioned that diazoxide during its presence in high concentrations decreases mitochondrial membrane potential in beta cells [43]. It was also reported that diazoxide during its presence protects beta cells from streptozotocin toxicity [44]. Furthermore, in heart muscle diazoxide exerts direct effects on mitochondria which are coupled to some degree of protection against myocardial ischemia [45, 46]. Diazoxide may also have beneficial effects in other tissues [47]. It could be conjectured that diazoxide confers by as yet undefined mechanisms a heightened defense against oxidative stress which is demonstrable only in situations of increased oxidative stress; such as exposure to toxins and to hyperglycemia. However, studies by ourselves indicate that the beneficial effects of diazoxide that we observe in vitro can be mimicked by somatostatin, which inhibits insulin secretion by mechanisms totally different from those of diazoxide [48].

### FUTURE DIRECTIONS OF CLINICAL RESEARCH ON OVERSTIMULATION

There is general consensus that present therapies in type 2 diabetes are not sufficient to uphold good metabolic control in the course of the disease and that new therapeutic modalities are needed. As reviewed above, there is by now much experimental evidence linking over-stimulation with beta cell demise. Luckily from a therapeutic perspective the situation seems much more amenable to treatment than in the GK animal model of type 2 diabetes, in which the progression of diabetes may be genetically determined. In our view, the time is therefore ripe for new clinical studies which target the role of over-stimulation and the clinical importance of “beta cell rest” in type 2 diabetes. One way to achieve “beta cell rest” is to increase insulin sensitivity. This can be done pharmacologically with metformin and with the newly developed thiozoladindiones, such as rosiglitazone and pioglitazone. Compatible with this notion metformin can delay onset of type 2 diabetes [49]. Another way is to use drugs such as diazoxide in the treatment of type 2 diabetes. The clinical use of diazoxide is hampered by the drug’s side effects. However, the doses so far used may well be higher than those that would significantly “rest” beta cells and improve beta cell function. There is also the possibility that analogues of diazoxide could produce lesser side effects. At least one such analogue is presently undergoing testing [44].

Lastly it should be pointed out that any intervention to improve insulin secretion should start early in the disease when endogenous insulin secretion (and presumably the number of functional beta cells) has not decreased too far. Evidence indicates that it would be far too late to await the stage when metabolic control has deteriorated to the point where insulin supplementation is considered, in which case the possibility of improving beta cell function by any means is very limited [50].

### REFERENCES

[1] Hamman, R. (1992) Genetic and environmental determinants of non-insulin-dependent diabetes mellitus (NIDDM). *Diabetes Metab. Rev.,* **8,** 287–338.

[2] Hattersley, A. (1998) Maturity-onset duiabetes of the young clinical heterogeneity explained by genetic heterogeneity. *Diabetic Medicine,* **15,** 15–24.

[3] UK Prospective Study (1995) Overview of 6 years therapy of type 2 diabetes: A progressive disease. UK Prospective Diabetes Study Group. *Diabetes,* **44,** 1249–1258.

[4] Clauson, P., Linnarsson, R., Sundkvist, G., Gottsäter A., and Grill V. ( 1994) Relationship between diabetes control and beta-cell function in a representative population of NIDDM subjects in Sweden. *Diabetic Medicine.,* **11,** 794–801.

[5] Kosaka, K., Kuzuya, T., Akimura, Y., and Hagura, R. (1980) Increase in insulin response after treatment of overt maturity-onset diabetes independent of the mode of treartment. *Diabetologia,* **18,** 23–28.

[6] Vague, P., and Moulin, J. P. (1982) The defective glucose sensitivity of the B cell in non insulin dependent diabetes. Improvement after twenty hours of normoglycaemia. *Metabolism,* **31,** 139–422.

[7] Glaser, B., Leibovich, G., Nesher, R., Hartling, S., Binder, C., and Cerasi, E. (1988) Improved beta-cell function after intensive insulin treatment in severe non-insulin-dependent diabetes. *Acta Endocrinol (Copenh),* **118,** 365–373.

[8] Clausson, P., Alvarsson, M., and Grill, V. (1997) Enhancement of $\beta$-cell secretion by blood glucose normalization relates to fasting C-peptide levels. *J. Internal Medicine,* **241,** 493–500.

[9] Grill, V., and Björklund, A. ( 2000) Dysfunctional insulin secretion in type 2 diabetes: Role of metabolic abnormalities. *Cell. Mol. Lifes Sci.,* **57,** 429–440.

[10] Cerami Buccala, R., Cerami, A., and Vlassara, H. (1995) Advanced glycosylation end products in diabetic complications. *Diabetes Reviews,* **3,** 258–268.
[11] Baynes, J., and Thorpe, S. (1999) Role of oxidative stress in diabetic complications. A new perspective on an old paradigm. *Diabetes,* **48,** 1–9.
[12] Tajiri, Y., Möller, C., and Grill, V. (1997) Long term effects of aminoguanidine on insulin release and biosynthesis. Evidence that the formation of advanced glycosylation end products inhibits B-cell function. *Endocrinology,* **138,** 273–280.
[13] Tanaka, Y., Gleason, C., Tran, P., Harmon, J., and Robertson, P. (1999) Prevention of glucose toxicity in HIR-T15 cells and Zucker diabetic fatty rats by antioxidants. *Proc. Natl. Acad. Sci. USA,* **96,** 10857–10862.
[14] Kaneto, H., Kajimoto, Y., Miyagawa, J., Matsuoka, T., Fujitani, Y., Umayahara, Y., Hanafusa, T., Matsuzawa, Y., Yamasaki, Y., and Hori, M. (1999) Beneficial effects of antioxidants in diabetes: Possible protection of pancreatic beta-cells against glucose toxicity. *Diabetes,* **48,** 2398–2406.
[15] Leahy, J., Cooper, H., Deal, D., and Weir, G. (1986) Chronic hyperglycemia is asssociated with impaired glucose influence on insulin secretion. A study in normal rats using chronic in vivo glucose infusions. *J. Clin. Invest.,* **77,** 908–915.
[16] Sako, Y., and Grill, V. (1990) Coupling of B-cell desensitization by hyperglycemia to excessive stimulation and circulating insulin in glucose-infused rats. *Diabetes,* **30,** 1580–1583.
[17] Björklund, A., and Grill, V. (1993) B-cell insensitivity in vitro: Reversal by diazoxide entails more than one event in stimulus-secretion coupling. *Endocrinology,* **132,** 1319–1328.
[18] Leahy, J., Bumbalo, L., and Chen, C. (1994) Diazoxide causes recovery of beta-cell glucose responsiveness in 90% pancreatectomized diabetic rats. *Diabetes,* **43,** 173–179.
[19] Hiramatsu, S., Höög, A., Möller, C., and Grill, V. (2000) Treatment with diazoxide causes prolonged improvement of beta-cell function in rat islets transplanted to a diabetic environment. *Metabolism,* **49,** 657–661.
[20] Goto, Y., Kakizaki, M., and Masaki, N. (1975) Spontaneous diabetes produced by selective breeding of normal Wistar rats. *Proc. Jpn. Acad.,* **51,** 80–85.
[21] Björklund, A., Östenson, C.-G., and Grill, V. (1997) Defective insulin secretion in the GK rat is not linked to excessive B-cell stimulation. *Pancreas,* **4,** 212–214.
[22] Suzuki, N., Aizawa, T., Asanuma, N., Sato, Y., Komatsu, M., Hidaka, H., Itoh, N., Yamauchi, K., and Hashizume, K. (1997) An early intervention accelerates pancreatic beta-cell dysfunction in young Goto-Kakizaki rats, a model of naturally occuring non-insulindependent diabetes. *Endocrinology,* **138,** 1106–1110.
[23] Björklund, A., and Grill, V. (1999) Enhancing effects of long-term elevated glucose and palmitate on stored and secreted proinsulin-to-insulin ratios in human pancreatic islets. *Diabetes,* **48,** 1409–1415.
[24] Leahy, J. (1993) Increased proinsulin/insulin ratio in pancreas extracts of hyperglycemic rats. *Diabetes,* **42,** 22–27.
[25] Laedtke, T., Kjems, L., Porksen, N., Schmitz, O., Veldhuis, J., Kao, P., and Butler, P. (2000) Overnight inhibition of insulin secretion restores pulsatility and proinsulini/insulin ratio in type 2 diabetes. *Am. J. Physiol.,* **279,** E520–E528.
[26] Ward, W. K., LaCava, E. C., Paquette, T. L., et al. (1987) Disproportionate elevation of immunoreactive proinsulin in type 2 (non-insulin-dependent) diabetes mellitus and in experimental insulin resistance. *Diabetologia,* **30,** 698–702.
[27] Seaquist, E., Kahn, S., Clark, P., et al. (1996) Hyperproinsulinemia is associated with increased beta cell demand after pancreatectomy in humans. *J. Clin. Invest.,* **97,** 455–460.
[28] Grill, V., Dinesen, B., Carlsson, S., Efendic, S., Pedersen, O., and Östenson, C.-G. Hyperproinsulinemia and proinsulin to insulin ratios in Swedish middle-aged men: Association with glycemia and insulin resistance but not with family history of diabetes. *Am. J. Epidemiology*. In press.
[29] Greenwood, R., Mahler, F., and Hales, C. (1976) Improvment of insulin secretion in diabetes after diazoxide. *Lancet,* **1,** 444–447.
[30] Olzak, S., Greenwood, R., and Hales, C. (1986) Post-receptor insulin resistance after diazoxide in non-insulin dependent diabetes. *Horm. Metabol. Res.,* **18,** 38–41.
[31] Björk, E., Berne, C., Kämpe, O., Wibell, L., Oskarsson, P., and Karlsson, A. (1996) Diazoxide treatment at onet preserves residual insulin secretion in adults with autoimmunne diabetes. *Diabetes,* **45,** 1427–30.
[32] Lillioja, S., Mott, D. M., Spraul, M., Ferraro, R., Foley, J. E., Ravussin, E., Knowler, W. C., Bennett, P. H., and Bogardus, C. (1993) Insulin resistance and insulin secretory dysfunction as precursors of non-insulin-dependent diabetes mellitus. Prospective studies of Pima Indians. *N. Eng. J. Med.,* **329,** 1988–1992.
[33] Everhart, J., Pettitt, J., Bennet, P., and Knowler, W. (1992) Duration of obesity increases the incidence of NIDDM. *Diabetes,* **41,** 235–240.
[34] Carlsson, S., Perssson, P.-G., Grill, V., Alvarsson, M., Efendic, S., Norman, A., Svanström, L., and Östenson C.-G. (1998) Weight history, glucosae intolerance and insulin levels in Swedish middle-aged men. *Am. J. Epidemiol.,* **148,** 539–545.
[35] Efanova, I., Zaitsev, S., Zhivotovsky, B., Köhler, M., Efendic, S., Orrenius, S., and Bergren, P.-O. (1998) Glucose and tolbutamide induce apoptosis in pancreatic beta cells. *J. Biol. Chem.,* **273,** 33501–7.
[36] Alvarsson, M., Berntorp, K., Forbes, E., Grill, V., Lager, L., Steen, L., Sundkvist, G., and Örn, T. (2001) Beneficial effects of insulin vs. sulphonylurea treatment early in type 2 diabetes on stimulated C-peptide and metabolic control. *Diabetologia,* **44**(Suppl 1), 216 (Abstract).
[37] Leahy, J. (1996) Beta-cell dysfunction with chronic hyperglycemia: "overworked betacell" hypothesis. *Diabetes Rev.,* **4,** 298–319.
[38] Renström, E., Eliason, L., Bokvist, K., and Rorsman, P. (1996) Cooling inhibits exocytosis in single mouse pancreatic B-cells by suppression of granule mobilisation. *J. Physiol.,* **494,** 41–52.
[39] Klöppel, G., Löhr, M., Habich, K., et al. (1985) Islet pathology and the pathogenesis of type 1 and type 2 diabetes mellitus revisited. *Surv. Synth. path. Res.,* **4,** 110–125.
[40] Kahn, S., Andrikopolous, S., and Verschere, C. (1999) Islet amyloid. A long-recognized but underappreciated pathological feature of type 2 diabetes. *Diabetes,* **48,** 241–253.
[41] Andrikopoulos, S., Verschere, B., Terauchi, Y., Kadowaki, T., and Kahn, S. (2000) Beta-cell glukokinase deficiency and hyperglycemia are associated with reduced islet amyloid deposition in a mouse model of type 2 diabetes. *Diabetes,* **49,** 2056–2062.
[42] Trube, G., Rorsman, P., and Ohno-Shosaku, T. (1986) Opposite effects of tolbutamide and diazoxide on the ATP-dependent potassium channel in mouse pancreatic B-cells. *Pflugers Arch.,* **407,** 493–499.

[43] Grimmsmann, T., and Rustenbeck, I. (1998) Direct effects of diazoxide on mitochondria in pancreatic B-cells and on isolated liver mitochondria. *Brit. J. Pharmacol.,* **123,** 781–788.

[44] Kullin, M., Zhanchun, L., Bondo Hansen, J., Björk, E., Sandler, S., and Karlsson, A. (2000) K-ATP channel openers protect rat islets against the toxic effect of streptozotocin. *Diabetes,* **49,** 1131–1136.

[45] Iwai, T., Tanonaka, K., Koshimizu, M., and Takeo, S. (2000) Preservation of mitochondrial function by diazoxide during sustained iscemia of the heart. *Br. J. Pharmacol.,* **129,** 1219–1227.

[46] Xu, M., Wang, Y., and Ashraf, M. (2001) Mitochondrial K(ATP) channel activation reduces anoxic injury by restoring mitochondrial membrane potential. *Am. J. Physiol.,* **281,** 295–303.

[47] Chi, X., Sutton, E., Hellerman, G., and Price, J. ( 2000) Potassium channel openers prevent beta- amyloid toxicity in bovine vascular endothelial cells. *Neuroscience Letters,* **290,** 9–12.

[48] Björklund, A. (1999) Effects of over-stimulation by glucose on pancreatic beta cell functioning: Studies in vitro with diazoxide. Thesis.Stockholm ReproprintAB.

[49] Knowler, W. C., Barrett-Connor, E., Fowler, S. E., Hamman, R. F., Lachin, J. M., Walker, E. A., and Nathan, D. M. (2002) Diabetes Prevention Program Research Group. Reduction in the incidence of type 2 diabetes with lifestyle intervention or metformin. *N. Eng. J. Med.,* **346,** 393–403.

[50] Kärvestedt, L., Andersson, G., Efendic, S., and Grill, V. (2002) A rapid increase in beta-cell function by multiple insulin injections in type 2 diabetic patients is not further enhanced by prolonging treatment. *J. Int. Med.,* **251,** 307–316.

